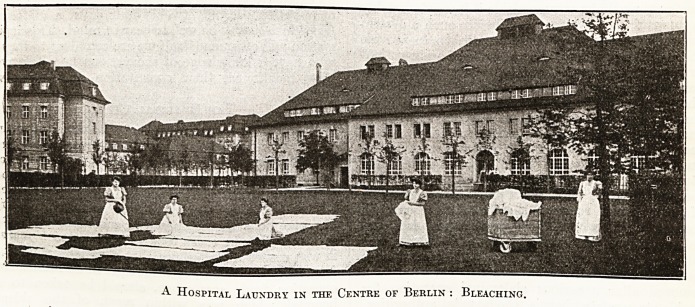# Government (Teaching) and Municipal (Non-Teaching) German Hospitals


**Published:** 1912-12-14

**Authors:** Henry Burdett


### GOVERNMENT (Teaching) and MUNICIPAL (Non-Teaching) GERMAN HOSPITALS.*

By SIR HENRY BURDETT, K.C.B.

### IX. British and German Hospitals: Some Contrasts.

Later on we propose to deal with the actual nursing system, if it can be called a system, in German hospitals; with the planning and administration of the city hospitals; and with other departmental matters of importance. In this article we wish as a preliminary to deal more generally with a few of the marked contrasts between a British voluntary hospital and one of the larger municipal or Government hospitals in Germany. One marked feature which characterises the best of all the German hospitals is the amount of attention which is given to the garden. By this we mean the surprising care, the enterprise exhibited in making the whole of the surroundings of each pavilion and the hospital grounds as a whole as attractive and health-giving as possible. Dr. Goldwater, speaking as we assume from what he regards as the point of view of municipalities and city residents in great cities in the United States, sets out to condemn the pavilion type of hospital, with its extensive gardens and attractiveness, by characterising some of the best of German hospitals as "text-book hospitals," and attributes their existence to "the unhygienic conditions and the undeveloped sanitary science of forty or fifty years ago." No one who has spent sufficient time in making a careful inspection and study of the best type of German city hospital, with its gardens and health-giving surroundings, being a just man, could support the view expressed by this American critic. The best type of the German hospitals of to-day had no existence in fact forty or fifty years ago. They are the outcome of the National Insurance Acts; and the oldest of them is under thirty years, whereas the best and most recent have been planned and erected within the last fifteen and some of the best within the last ten years. The German municipalities, too, have had the most competent advice, and have exercised infinite care, regardless of expense, in the selection of the site and grounds for a large hospital in their important cities. The first considerations applying to such a site in the German scientist's view are, properly, that it must be chosen with the greatest care; that it must be withdrawn from the neighbourhood of the factories causing noise, smoke, and soot; that it must be as open as possible, having regard to distance from the interior of the city it is to serve; and, not the least important of all, that it must have considerable dimensions in order to provide room for sufficiently large gardens. The maintenance of these gardens and the selection of such a site have a material bearing upon the earning powers of the workers in great cities throughout Germany. This is so, because where the conditions just referred to are fulfilled in the choice of a site for a city hospital, there the workman or workwoman who has to undergo a serious operation can be treated under conditions, which hygienically will guarantee, with a minimum residence, a maximum of facility to ensure the speedy return of the worker to his occupation in a state of health which will enable him to take it up promptly on his discharge from the hospital. We will not enter into the question of plans here, except to say that, whereas the German hospitals of the best type with their grounds and gardens do fulfil these conditions admirably on the whole, the heap-of-buildings type without any grounds whatever, consisting as it may do of one immense block of buildings soaring skyward floor above floor, cannot, however efficient the medical and surgical treatment may be, enable patients, immediately on their discharge from such a hospital,

A Hospital Laundry in the Centre of Berlin: Bleaching.

* Previous articles of this series appeared in our issues of Oct. 12, 19, 26, Nov. 2, 9, 23, 30, and Dec. 7.

to take up their work with full energy and without the risk of again breaking down in health. For this reason alone, if any regard is to be paid to the humanitarian aims and objects which ought to govern the administration of a great town hospital for the sick, there can be no doubt that the more intelligent, humane, and knowledgeable the inhabitants of any city may be, the greater the guarantees that the one great block or the heap-of-buildings type of hospital will find no place in the centre of an up-to-date, really unselfish, and thoughtful city community. This is not a question of race, or creed, or nation. It is simply a case of the individual standard which animates the people of all classes in a great city in the discharge of their duty towards their neighbour and towards the prosperity, popularity, and success of the town in which they reside.

### The Average Stay of In-Patients.

In England, especially of late years, there has been a trend towards the shortening of the residence of in-patients, and especially of surgical in-patients, in some of the larger and older hospitals in cities. This tendency would be commendable if it was invariably accompanied by evidence that before such a system was adopted every care had been taken to provide that this shortening of the residence of an in-patient in a hospital, situated in a great city, was accompanied by a system which provided that, on discharge, every such patient should be removed to a semi-convalescent hospital, outside the city boundaries, and placed under health conditions which secured to him the best of air, and the most restful and beneficial influences in the shape of gardens and atmosphere. Where this shortened residence has merely meant the discharge of patients in a condition of health which rendered them unfit to attempt to resume work, where their needs demanded convalescent care for a fortnight or three weeks, and where, as in a case which came under our notice only this week, all the stitches had not even been removed after a serious abdominal operation for double strangulated hernia, the hospital authorities, to whatever nation they may belong, should be held responsible to the public for the almost certain disastrous consequences which must ensue for the unfortunate patient, suffering from premature dismissal from the care of the hospital authorities. We have heard some of the authorities of the larger hospitals in this country claim that their cases are necessarily retained for a longer time in the wards than those of a neighbouring hospital which is competing keenly with it for the support of the public. By necessarily retained they mean that as conscientious men they cannot and will not suffer any pressure upon the beds to lead them, or to suffer them to permit any official attached to the hospital, to discharge a patient, unless to a convalescent home, who is not in a condition of health or circumstances to resume his work, or at least to be placed when at home under conditions which will speedily build up his constitution and enable him to go about his ordinary vocations. In Germany, to avoid dangers of this kind and to meet as far as possible the pressing needs of workers in great cities who become in-patients of a city hospital, the gardens and ample grounds with the additional cost entailed have been cheerfully faced, so that there shall be no thought of a patient being sent out to work before he is in a physical condition to undertake it. In British clinical hospitals the average stay of each patient has been reduced from some thirty-five days to twenty days or less. In Germany, speaking of municipal hospitals, the average stay is some twenty-seven days and sometimes longer. For instance, according to the last returns, it was thirty-one days at the Johannstadt Hospital, Dresden; thirty days at the City Hospital, Carlsruhe; twenty-nine days at Elberfeld and at the Charlottenburg Hospital, Berlin; and twenty-eight days at the Town Hospital, Düsseldorf, and the St. Jakob Hospital, Leipziz. On the other hand, the number of days residence has been reduced to twenty-two days at Hanover, to twenty-three days at Danzig, whilst it is twenty-six days at Breslau, at Cologne (Lindenberg), at Hamburg (St. Georg), and at Kiel. In contrast to the longer days of residence we may mention that at the Rudolf Virchow Hospital, Berlin, with its extensive grounds and gardens, the average stay of in-patients is only twenty-four days, a material fact which it may be well to bear in mind. This intimation will commend itself we are confident to those who have a knowledge of the various hospitals we have mentioned, their constitution and administration.

### The Hospital City.

In our view the best of the German hospitals, with their large gardens and attractive grounds, are but an intermediate step in the development of hospital construction upon lines which the increasing needs of the population of great cities all the world over must ultimately result. Until the enormous developments in mechanical means of locomotion in cities took place in recent years, it was essential, to secure that a patient suffering from serious accident, or who required an immediate operation from any cause, should be taken to a hospital which was within reach of the ablest members of the surgical staff. To-day it is relatively immaterial as to whether the hospital be a mile or even ten miles from the residence of the great surgeon. To-day a hospital service with a well-organised system of motor ambulances would bring a hospital situated outside the city boundaries, in the best of atmosphere and under the most perfect hygienic conditions, within the ready reach of the best medical and surgical aid in the cities. Another factor which materially affects the modern hospital is that the ever-increasing number of people of all classes, who desire hospital care when seriously ill, makes it essential that each hospital shall provide adequate accommodation for all classes of the community, and that the hospital buildings shall include worthy house accommodation as resident quarters for an able, up-to-date, and a perfectly efficient medical staff, capable of handling promptly and adequately every case which may be brought for treatment. Again, the association together of hospital pavilions upon the same site to afford accommodation for all classes of citizens, from the highest to the humblest, means that the site of the hospitals must in future be selected to secure the very best available area, within reasonable distance from the heart of the city. The extent of that distance must, however, depend largely upon the staple trades of the particular city and the surrounding district. Where all classes of the citizens have a vital interest in the utmost efficiency and the very finest equipment of the city hospital, there should certainly be found the best of everything which modern science and the best of treatment demands as aids to the rapid recovery of each patient within its walls. So we venture to remind every one interested in the subject, that the ideal of a Hospital City, which has been so fully elaborated in the article on Hospitals in the new edition of the "Encyclopædia Britannica," is one which is well calculated to commend itself to the judgment of those cities, at any rate, where the administration of the city affairs is governed by the most commanding intelligence and fulness of knowledge.

Semi-Convalescent Homes.

In England, as in the case of the General Infirmary at Leeds, for example, to quote one instance out of many to be found in this country, two semi-convalescent hospitals, the Ida and Robert Arthrington at Cookridge, have been opened with excellent results. Semi-convalescent in plain English is a receiving house for in-patients with open wounds, who have been operated upon in a city hospital and have arrived at a stage on the road to recovery, when it is manifest that to hasten a cure and complete it rapidly and with the utmost efficiency a better quality of air than that the city affords is demanded in the patients' interests. We should be glad to hear from the United States to what extent, if at all, provision has been made by semi-convalescent hospitals to help the workers treated as in-patients in the large city hospitals there to a rapid and complete recovery after operations for serious illness. In Germany convalescent institutions were practically unknown in the British sense until comparatively recent years, and it is quite possible, as the Kassen and public opinion are evidently moving in the direction of more and more convalescent institutions, that in the great city hospitals which may yet have to be built some attention may be paid to the idea of semi-convalescent hospitals and their uses. We should, however, hope, having regard to the extraordinary efficiency with which the German mind attacks the hygienic and scientific aspect of every question, that, benefiting by the experience of England and other nations, the fathers of some great city will take their courage in both hands and determine to erect, under the ægis of a great municipality, a Hospital City outside the corporation boundaries. It has always surprised us that that home of stupendous enterprises, the United States of America, has not yet grasped, put into practice, and made a great practical object-lesson the ideal of the Hospital City. For in that country, where great cities are apt to spring up in a year, such an ideal could find practical expression with the maximum of speed, economy, and success.

*(To be continued.)*

## Figures and Tables

**Figure f1:**